# High-Altitude Genetic Selection and Genome-Wide Association Analysis of Yield-Related Traits in *Elymus sibiricus* L. Using SLAF Sequencing

**DOI:** 10.3389/fpls.2022.874409

**Published:** 2022-06-21

**Authors:** Zongyu Zhang, Yuying Zheng, Junchao Zhang, Na Wang, Yanrong Wang, Wenhui Liu, Shiqie Bai, Wengang Xie

**Affiliations:** ^1^The State Key Laboratory of Grassland Agro-Ecosystems, Key Laboratory of Grassland Livestock Industry Innovation, Ministry of Agriculture and Rural Affairs, College of Pastoral Agriculture Science and Technology, Lanzhou University, Lanzhou, China; ^2^Institute of Qinghai-Tibetan Plateau, Southwest Minzu University, Chengdu, China; ^3^Key Laboratory of Superior Forage Germplasm in the Qinghai-Tibetan Plateau, Qinghai Academy of Animal Science and Veterinary Medicine, Xining, China; ^4^Sichuan Academy of Grassland Science, Chengdu, China

**Keywords:** *Elymus sibiricus*, genetic signal selection, GWAS, high altitude, multi-algorithms analysis, yield-related traits, hub genes

## Abstract

The genetic adaptations to harsh climatic conditions in high altitudes and genetic basis of important agronomic traits are poorly understood in *Elymus sibiricus* L. In this study, an association population of 210 genotypes was used for population structure, selective sweep analysis, and genome-wide association study (GWAS) based on 88,506 single nucleotide polymorphisms (SNPs). We found 965 alleles under the natural selection of high altitude, which included 7 hub genes involved in the response to UV, and flavonoid and anthocyanin biosynthetic process based on the protein–protein interaction (PPI) analysis. Using a mixed linear model (MLM), the GWAS test identified a total of 1,825 significant loci associated with 12 agronomic traits. Based on the gene expression data of two wheat cultivars and the PPI analysis, we finally identified 12 hub genes. Especially, in plant height traits, the top hub gene (TOPLESS protein) encoding auxins and jasmonic acid signaling pathway, shoot apical meristem specification, and xylem and phloem pattern formation was highly overexpressed. These genes might play essential roles in controlling the growth and development of *E. sibiricus*. Therefore, this study provides fundamental insights relevant to hub genes and will benefit molecular breeding and improvement in *E. sibiricus* and other *Elymus* species.

## Introduction

*Elymus* is the largest and most widely distributed genus of Triticeae (Poaceae) with a valuable gene pool that can be used for the genetic improvement of cereal crops such as wheat (*Triticum aestivum* L.), barley (*Hordeum vulgare* L.), and rye (*Secale cereale* L.) due to its excellent stress tolerance, disease resistance, and good forage quality (Dewey, 1984; Ma et al., 2012a). Siberian wild rye (*Elymus sibiricus* L.), the type species of the genus *Elymus*, is a perennial, sparse-type, self-pollinating, and allotetraploid grass (2*n* = 4× = 28). *Elymus sibiricus* is commercially useful in stock raising and serves as a major forage grass in natural and artificial grasslands. In addition, it is widely being used in the restoration of ecosystems, soil stabilization, and erosion control due to its good ecological function (Xie et al., 2015).

Natural selection and artificial domestication in plants have a pivotal role in reshaping the genome, promoting the evolution of populations, high allele frequency in selective sweep regions, local adaptation, and genetically based trait differentiation (Chapman et al., 2013). The geographic distribution of wild *E. sibiricus* accessions mainly extended from Eastern Europe to Siberia, Mongolia, China, Japan, and even to Alaska and Canada, leaving extensive genetic variations and evolutionary signatures in the genome (Ma et al., 2012a). The Qinghai-Tibet Plateau (QTP) is a hot spot area for studying genetic adaptations, as the average elevation of about 4,000 m above sea level caused the extreme ecological conditions, such as high UV-B radiation and low oxygen concentration (∼30% stronger and ∼40% lower than at sea level, respectively; Yang et al., 2017). The genetic basis of harsh climatic adaptations in high-altitude regions has been reported and many genome selective regions and strongly associated environmentally related genes were found for humans, animals, and plants (Li M. et al., 2013; Yang et al., 2017; Wang et al., 2021a,b). *E. sibiricus* grows on wet meadows, shrubs, forests, and subalpine meadows, where altitude gradients range from 0 to 4,000 m above the sea level, yet the selective signatures at molecular level between high-altitude and low-altitude habitats remain unclear.

Recently, increasing attention has been paid to the requirement of high-yield and high-quality forage varieties, such as *E. sibiricus* (Jin et al., 2021). The annual demand for hay and grass seeds in China exceeds 10 million and 150 thousand tons, respectively. However, traditional breeding and phenotypic selection still rely heavily on experience and manual observation, which is time consuming, low efficiency, and environmentally sensitive. The genome-wide association study (GWAS) is a powerful tool for the rapid identification of loci or key genes associated with agronomic traits by analyzing associations between nucleotide polymorphisms and phenotypic diversity. These associations were measured by using a natural or low-structured population, in which a large number of recombination events were found based on linkage disequilibrium (LD; Flint-Garcia et al., 2003). The abundant genetic variations from GWAS facilitate more targeted and accurate genetic selection for marker-assisted selection (MAS) and molecular design breeding when compared with quantitative traits loci (QTL) mapping. Recently, advances in next-generation high-throughput sequencing technologies provide a strategy for high-density genotyping based on large-scale populations at the genome-wide level. An increasing number of GWASs have been reported in crops and forage grasses for important agronomic traits such as heading date, plant height, and panicle length in rice (*Oryza sativa* L.; Yano et al., 2016); fiber quality and yield traits in cotton (*Gossypium hirsutum* L.; Fang et al., 2017a); spike ethylene production and spike dry weight in wheat (Valluru et al., 2017); drought adaptation in maize (*Zea mays* L.; Zhang et al., 2021); salt germination, frost damage, fall dormancy, and unifoliate internode length in alfalfa (*Medicago sativa* L.; Shen et al., 2020); and flowering time in switchgrass (*Panicum virgatum* L.; Grabowski et al., 2017). Limited by high-sequencing costs and difficulties in genome assembly, the genomic information of most species is still unknown, especially for polyploidy plants with large size and complex genomes. Sun et al. (2013) tested an efficient approach (specific-locus amplified fragment sequencing, SLAF-seq) for *de novo* single nucleotide polymorphism (SNP) discovery, namely reduced-representation genome sequencing (RRGS) technology. Several distinguishing characteristics have propelled much of the practicability of SLAF-seq including the generation of millions of high-density SNPs, improvement of efficiency and performance–cost ratio by missing repetitive sequences, capability of detecting novel SNPs compared with SNP arrays, and suitability for species without a reference genome. The technology has successfully applied to many species with haplotype analysis, linkage mapping, and genetic association studies (Li et al., 2017; Zhang et al., 2019a; Zhuo et al., 2021).

Although GWAS test has the potential to detect numerous genetic polymorphisms or QTLs associated with complex agronomic traits, it is difficult to completely avoid possible spurious loci caused by population stratification and unequal relatedness among the studied individuals (Kang et al., 2010; Zhang et al., 2010). The mixed linear model (MLM) has been proven effective as a useful method to control-inflated associations for principal components (or population structure) and kinship modeling as a fixed effect and random effect, respectively (Yu et al., 2006). However, the exact statistics test approach was computationally impractical in dealing with large data sets, for the iterative time of each SNP increased sharply with the number of individuals as a random effect. A P3D (population parameters previously determined) method implemented within the TASSEL (Trait Analysis by aSSociation, Evolution and Linkage) program to reduce the running time from standard MLM proposed an approximate algorithm, which replaced the repeated iteration of population parameters by fixed empirical Bayesian priors (Zhang et al., 2010). The statistical power of P3D has been maintained or improved regardless of the different genetic architectures of the phenotype. Similarly, Efficient Mixed-Model Association eXpedited (EMMAX) simplified the estimation of variance parameters once by using a null hypothesis, based on natural characteristics that the most effective of a single locus is small in complex quantitative traits (Kang et al., 2010). In practice, inaccurate *P* values may result from the approximation approach when dealing with highly structured populations and strongly associated marker effects. Contrarily, an exact association test was provided from GEMMA (Genome-wide Efficient Mixed Model Association) on which the Newton–Raphson optimization method was presented. GEMMA remained the identical results compared with the exact calculations in EMMA, but was roughly *n* times faster, where *n* was the sample size (Zhou and Stephens, 2012). As the GEMMA strictly needs completed or imputed genotype data sets (sample integrity > 0.95), some approximate algorithms remain necessary, especially in the absence of large-scale effective SNPs by using an RRGS. A previous research showed that a conjoint analysis of multiple models favored improving the accuracy and reducing the false positive rate. Fifty-seven QTLs related to seed size and oil content traits in soybean (*Glycine max* L.) were commonly found using three approaches: mrMLM, GEMMA, and EMMAX (Liu et al., 2020). A similar strategy was reported within maize, barley, flax (*Linum usitatissimum* L.), masson pine (*Pinus massoniana*), spruce (*Picea abies* L. Karst), and pig experiments (Manunza et al., 2014; Gyawali et al., 2017; Xie et al., 2018; Bai et al., 2019; Gao et al., 2019; Chen et al., 2021).

In this study, we performed a genome-wide SNP identification based on SLAF-seq of 210 *E. sibiricus* genotypes. Among these, the genetically selected loci and genes were examined by using two extreme groups from low and high altitudes. Further, the phylogenetic relationship, genetic architecture, and a GWAS of twelve grass yield- and seed-related traits were conducted. Our results provided a new genetic resource for molecular breeding in *E. sibiricus*.

## Materials and Methods

### Plant Materials and Field Trials

**FIGURE 1:**
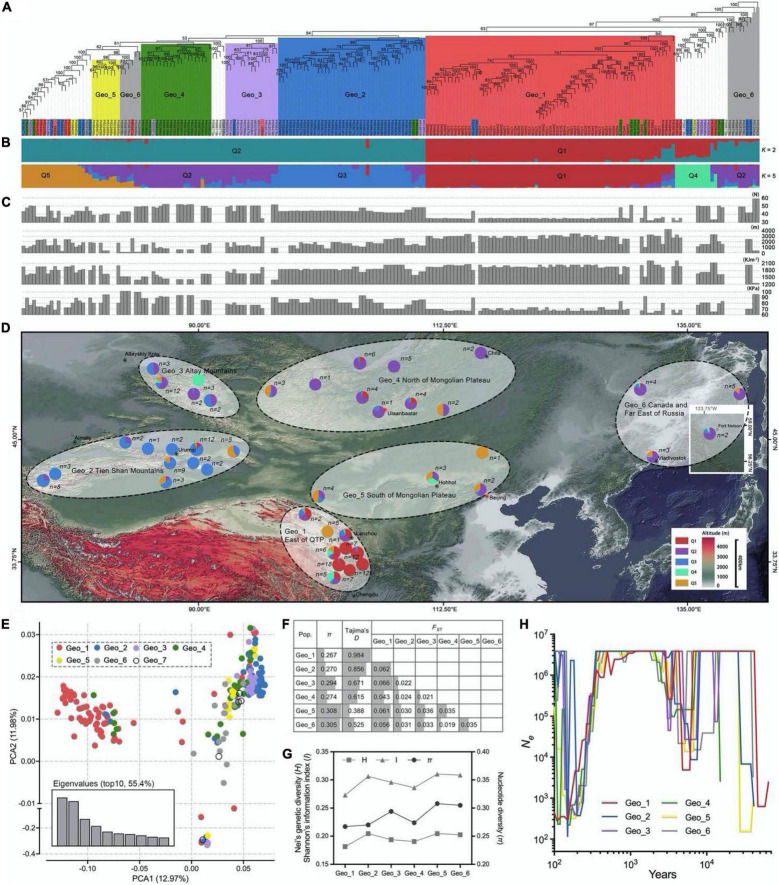
Population structure, geographic distribution, and genetic diversity analysis by using 88,506 high-consistent single nucleotide polymorphisms (SNPs). **(A)** The neighbor-joining (NJ) phylogenetic tree of 210 *Elymus sibiricus* individuals was constructed with 88,506 SNPs. The different colors, respectively, represent the six geographical populations. Only bootstrap values higher than 50% are presented. **(B)** Population genetic structure of *E. sibiricus* germplasms when the *K*-value was two and five. **(C)** Geographic and climatic information for each germplasm, including latitude, altitude, UV-B, and surface pressure. Each bar of **(B,C)** corresponded to every individual in **(A)**. **(D)** Geographical distribution of *E. sibiricus* samples. The pie chart in the map represents the genetic structure proportion of each accession based on the population structure analysis (*K* = 5). **(E)** Scatter plots of the first two principal components. **(F)** Genetic diversity analysis among six populations. **(G)** Genetic diversity relationships of expressed sequence tag-derived simple sequence repeat marker (EST-SSR)-based Nei’s genetic diversity *(H)* and Shannon’s information index *(I)*, as well as SNP-based nucleotide diversity (π) among six *E. sibiricus* populations. **(H)** Demographic histories of wild *E. sibiricus* groups. Data are shown the estimates of effective population size (*N*_*e*_) from about 70 Kya for the six groups.

The 210 *E. sibiricus* genotypes used in this study were obtained from the Lanzhou University and the U.S. Department of Agriculture Germplasm Resources Information Network (GRIN). The latitude and altitude of the collection site ranged from 32.1°N to 58.8°N and from 0 to 4,300 m, respectively (Figures 1C,D and Supplementary Table 1). According to diverse geographical origin, these genotypes were divided into seven groups, including the East of Qinghai-Tibet Plateau group (Geo_1, 76 samples), Tien Shan Mountains group (Geo_2, 52 samples), Altay Mountains group (Geo_3, 22 samples), North of Mongolian Plateau group (Geo_4, 30 samples), South of Mongolian Plateau group (Geo_5, 11 samples), Canada and Far East of Russia group (Geo_6, 14 samples), and Unknown group (Geo_7, 5 samples).

The seedlings of all genotypes were planted for phenotypic investigation at Yuzhong experimental field of Lanzhou University (35.95°N, 104.15°E, altitude 1,720 m). Each individual was planted with 50-cm spacing in a randomized design. Twelve agronomic traits, including flag leaf length (FLL), flag leaf width (FLW), leaf length (LL), leaf width (LW), plant height (PH), culm diameter (CD), tiller number (TN), culm node number (CN), spike length (SL), length of seed (LS), width of seed (WS), and 1,000-seed weight (SW1) were assessed from 2017 to 2018. A total of 10 replications for each trait (except PH and TN) in an individual plant were recorded using the method described by the previous study (Zhang et al., 2018). The R package “corrplot” (v0.92) was used for a pairwise correlation analysis. The Shannon’s diversity index (H’) of each trait was calculated as previously described by Zhang et al. (2018).

### Single Nucleotide Polymorphism Genotyping

The genomic DNA of each genotype was extracted from young leaf tissues using the Qiagen DNeasy 96-well procedure (QIAGEN, Valencia, Calif). The quantity and quality of samples were measured using a Nano-Drop ND1000 spectrophotometer (NanoDrop, Wilmington, DE, United States). The final concentration of the DNA was adjusted to 50 ng/μl to meet the requirements of SLAF-seq (concentration ≥ 30 ng/μl, DNA quantity ≥ 2 μg).

*Elymus sibiricus* genome is not available; therefore, the wheat genome was selected as a reference genome^1^ (v1.0) for a higher genomic homology (79%) with *E. sibiricus* genetic linkage map (Zhang et al., 2019a). After the digestion prediction analysis of the reference genome, *Hae*III, the appropriate restriction enzyme, was used to digest the genomic DNA and to generate sequencing tags (SLAFs, approximately 464–494 bp in length). This step ensured that the obtained tags were evenly distributed among the chromosomes. The PCR, SLAF library construction, and paired-end sequencing were conducted as previously described (Sun et al., 2013; Zhang et al., 2019a). High-quality raw reads of each sample were mapped onto the reference genome to develop SLAFs by using the Burrows-Wheeler Aligner (BWA) software (v0.7.17; Li and Durbin, 2009). The Genome Analysis Toolkit (GATK, v4.2.0.0) was used for SNP calling (McKenna et al., 2010). The raw DNA sequencing data had been uploaded to NCBI (SRR17714927–SRR17715136). A total of 88,506 highly reliable population SNPs (missing data less than 0.5, minor allele frequency (MAF) greater than 0.05) were selected and used for subsequent analysis. The R package “CMplot” (v4.0.0) was used to show the distribution of SNPs in each chromosome.

### Population Structure, Genetic Diversity, and Selective Sweep Analysis

A neighbor-joining phylogenetic tree was constructed using PHYLIP with 500 bootstrap replicates^2^ (v3.698) and visualized on the online tool iTOL.^3^ The population structure of the tested natural population was assessed by the ADMIXTURE software (v1.3.0; Alexander et al., 2009). The optimum number of subgroups was determined according to cross-validation of the error rate with the presented *K*-value ranging from 1 to 10. A principal component analysis (PCA) was performed using PLINK^4^ (v1.90). In addition, the kinship matrixes among all genotypes were calculated by using GEMMA (v0.98.1) and then visualized by the “pheatmap” package (v1.0.12) in R (Zhou and Stephens, 2012). Nucleotide diversity (π), Tajima’s *D* value, and genetic differentiation (*F*_ST_) for each group were measured with VCFtools (v0.1.16; Danecek et al., 2011). The past evolution of the effective population size (*N*_*e*_) was estimated by using SMC + + (v1.15.4; Terhorst et al., 2016). A mutation rate of 1.3e-8 per synonymous site per generation was used. Two environmental data sets, surface UV-B radiation and surface pressure, were collected from European Centre for Medium-Range Weather Forecasts (ECMWF^5^, ERA5 monthly averaged data on single levels from 1979 to present) from 1979 to 2010 for the genome-wide selective sweep analysis on high-altitude group (greater than 3,000 m) and low-altitude group (less than 1,000 m). The selected SNPs were annotated to find related genes with response to UV-B and oxidative damage of UV radiation to plants among seven public databases: the NCBI non-redundant protein sequence (Nr, 202009^6^), Gene Ontology (GO, 20200615^7^), Protein family (Pfam, v33.1^8^), Cluster of Orthologous Groups (COG^9^), euKaryotic Orthologous Groups (KOG^10^), Annotated protein sequence database (Swiss-Prot, 202005^11^), and Kyoto Encyclopedia of Genes and Genomes (KEGG, 20191220^12^; Supplementary Table 4). The Search Tool for Retrieval of Interacting Genes/Proteins (STRING^13^ v11.5) was used to predict and construct the PPI network of the candidate genes based on the minimum required interaction score of medium confidence (Szklarczyk et al., 2019). The intersection genes with a high degree were regarded as candidate hub genes and the PPI network was visualized with the Cytoscape software (v3.8.2).

### Genome-Wide Association Study of Agronomic Traits

Three publicly GWAS statistical softwares, Genome-wide Efficient Mixed Model Association (GEMMA, v0.98.1), Efficient Mixed-Model Association eXpedited (EMMAX, beta-07Mar2010 version), and Trait Analysis by aSSociation, Evolution, and Linkage (TASSEL, v5.0), were used together to find out the underlying SNPs or genes associated with 12 grass yield-related traits in *E. sibiricus* (Bradbury et al., 2007; Kang et al., 2010; Zhou and Stephens, 2012). A MLM, with the first three PCA components and the kinship matrix considered a fixed effect and random effect, respectively, was implemented on these programs to carry out GWAS analysis. In addition, the SNP genotyping database was filtered more strictly (marker integrity frequency > 95%, MAF > 0.05) before the GEMMA analysis. Genome-wide significance thresholds were set as *P* < 1e-4 (EMMAX and TASSEL) and 1e-3 (GEMMA), whereas the Bonferroni-corrected cutoff was 0.1/total applied SNPs (approximately 1.13e-6 for EMMAX and TASSEL and 9.36e-6 for GEMMA; Supplementary Figures 3–5). The genes were located within 100-kb flanking regions around the significant trait-associated SNPs based on the public databases. All the genes were selected based on plant development process terms, including root, seed, leaf, flower, cell cycle, cell division, cell proliferation, as well as some related proteins, enzymes, hormones, transcription factors, and so on (Supplementary Table 6). The gene expression data from leaf-, culm-, and seed-related traits of two wheat cultivars (Azhurnaya and Chinese Spring) were collected from IWGSC RefSeq Annotations v1.0^14^ and used to analyze the gene expression profiles of each identified gene. The candidate genes were identified with an expression value (TPM) of more than 10. In addition, the non-synonymous variations that happened within the gene exon areas were used for the analysis of statistical differences for traits. The PPI network analysis was employed to remove the noise information and focus on the hub genes related to plant growth and development by using the STRING and Cytoscape softwares.

## Results

### Sequencing and Genome-Wide Polymorphic Single Nucleotide Polymorphism Identification

Using a SLAF high-throughput sequencing approach, a total of 1,176.09 million paired-end reads were generated from the raw sequence data from the panel of 210 *E. sibiricus* samples (Supplementary Table 2). The average GC (guanine-cytosine) contents were 45.88% and the Q30 percentage was 93.66%. A bioinformatics analysis aligned to the wheat reference genome revealed that the mapping rate of reads was ranged from 72.83 to 87.92%, with an average of 84.67%. Furthermore, 750,108 SLAF tags were identified with an average mapping depth per sample of 18.01x. A total of 13,643,515 SNP markers were generated. Upon filtering the genotype results with MAFs of 0.05 and sample locus integrity of 0.5, a total of 88,506 highly consistent SNPs were retained. The mean integrity and heterozygosity rate for each sample were 72.39 and 0.2271%, respectively. These obtained SNP markers were not evenly distributed on the genomic regions of 22 chromosomes of wheat (Table 1 and Supplementary Figure 1). The largest number and density of SNPs (6,704.6 and 84.2 kb/SNP) were on chromosome D, followed by chromosome B (3,395.6 and 220.1 kb/SNP) and chromosome A (2,332.0 and 314.2 kb/SNP). The mean number of SNPs was 4,023, ranging from 7,923 (chr7D) to 1,481 (chrUn), and the mean density per marker was 211.5 kb, ranging from 80.4 (chr6D) to 361.1 (chr4A).

**TABLE 1:** Distribution and summary of *E. sibiricus* genome-wide variants.

Chr^a^	SLAF number	Polymorphic SLAF	Number of SNPs	Number of SNPs after filtering	Max gap (kb)	Density of SNPs (kb/SNP)
chr1A	23,793	10,235	415,929	1,784	7,945	333.0
chr1B	30,204	13,794	551,062	3,020	12,875	228.4
chr1D	39,202	19,998	745,444	5,815	3,060	85.2
chr2A	31,014	13,938	556,863	3,641	8,880	214.4
chr2B	36,441	16,838	678,080	3,720	8,337	215.4
chr2D	50,737	25,978	968,703	7,480	5,039	87.1
chr3A	28,789	12,631	509,555	2,286	13,917	328.5
chr3B	38,882	17,580	664,931	3,627	11,223	229.1
chr3D	47,808	24,410	913,125	6,907	6,713	89.1
chr4A	28,344	12,405	500,044	2,062	12,589	361.1
chr4B	29,720	14,423	501,873	3,803	8,279	177.1
chr4D	37,143	19,032	689,559	6,020	4,898	84.7
chr5A	27,713	11,993	491,348	2,018	13,412	351.7
chr5B	33,734	15,698	618,957	3,630	6,587	196.5
chr5D	44,405	22,673	858,003	6,887	6,820	82.2
chr6A	23,461	10,219	413,822	1,827	13,586	338.3
chr6B	30,927	14,403	561,932	2,804	11,922	257.1
chr6D	35,973	18,356	685,631	5,891	6,193	80.4
chr7A	30,002	13,253	547,319	2,706	10,635	272.2
chr7B	33,044	15,114	598,225	3,165	8,661	237.2
chr7D	50,656	25,855	973,007	7,932	4,456	80.5
chrUn Mean	18,116 34,096	5,127 16,089	200,103 620,160	1,481 4,023	44,055 10,458.3	324.8 211.5
Total	750,108	353,953	13,643,515	88,506	–	–

*^a^Chromosome.*

### Population Structure, Genetic Diversity, and Selective Sweep Analysis

The kinship analysis showed that no direct genetic relationships between each two *E. sibiricus* samples in this study, for the kinship coefficients of more than 97.5% paired individuals, were less than.05 (Supplementary Figure 2). Phylogenetic relationships among 210 *E. sibiricus* genotypes were quantified using 88,506 high-quality SNPs (Figure 1A). The results showed a monophyletic branch in the neighbor-joining (NJ) tree, indicating a single domestication event happened among all samples. Most of the genotypes of Geo_1, Geo_2, Geo_3, Geo_4, and Geo_5 tended to be grouped according to their geographic origins, and the remains were formed in other admixture clusters. A model-based population structure analysis, reflecting additional inter- and intra-group relationships by the varying *K*-value (the number of ancestry kinship), had assigned the 210 genotypes into two and five subgroups (cross-validation errors of.498 and.521, respectively; Figure 1B). Different from others, genotypes that originated from the Qinghai-Tibet Plateau (Geo_1) had a close relationship when *K* was set to two (the best suitable number from the *K*-value test). When a higher *K*-value was assumed, more subgroups were further detected, including some groups from Tien Shan Mountains (Geo_2), Altay Mountains (Geo_3), and Mongolian Plateau (Geo_4 and Geo_5). Similar to the NJ tree, five structured populations (*K* = 5) were consistent with their geographical distribution characteristics (Figure 1D). The PCA mainly split the samples into two distinct groups (QTP and others) at an overall genomic level, in which the first two PCAs had explained 24.95% of the total variation (Figure 1E). A continuous distribution showed on the plot indicating no population stratification within the studied genotypes.

The nucleotide diversity (π value) was estimated for six major geographic groups, which was higher for Geo_3, Geo_5, and Geo_6 (average of.302) than Geo_1, Geo_2, and Geo_4 (average of.270; Figure 1F). These results were similar to our previous diversity study based on EST-SSR markers (Figure 1G; Zhang et al., 2019b). Positive mean Tajima’s *D* values existed within all of *E. sibiricus* groups which not only implied a lower probability of population expansion under the balancing selection but also less rare allele frequencies resided in specific habitats, especially in Geo_1 (0.984) and Geo_2 (0.856). The farthest genetic distance (average of.058) was inferred between 76 QTP samples and 129 genotypes from the other five groups using a genetic differentiation analysis (*F*_ST_). An evolutionary history analysis among different geo-populations showed similar demographic trajectories: all groups suffered bottleneck events that the effective population size (*N*_*e*_) decreased first and then increased to a stable size during 2–13 thousand years ago (Kya), and a short common history that the *N*_*e*_ size of 4E6 appeared in.4-2 Kya (Figure 1H). Thereafter, in the next bottleneck, effective population size significantly declined and then rebounded except for Geo_1 group.

**FIGURE 2:**
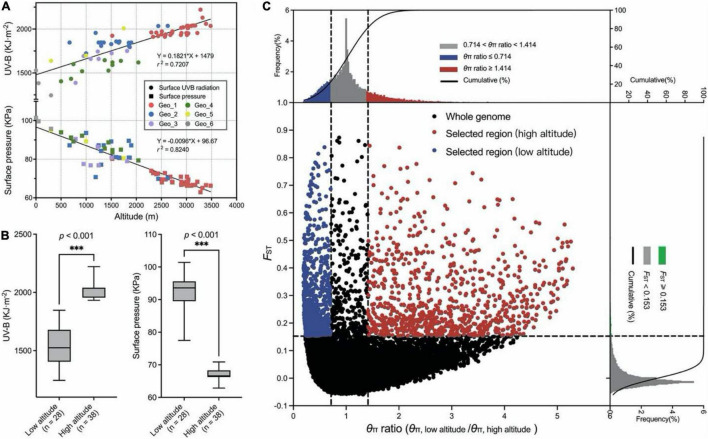
Selective sweep analysis. **(A)** Regression analysis between the UV-B, surface pressure, and altitude for 210 *E. sibiricus* genotypes. **(B)** Two boxplots for UV-B and surface pressure among low-altitude and high-altitude genotypes. Whiskers extend to data ranging from min to max values. Differences between the two pools were statistically analyzed based on the Welch’s test. **(C)** Distribution of θ_π_ ratio (θ_π_, low altitude/θ_π_, high altitude) and *F*_*ST*_ value. The horizontal dashed line represents the top 5% *F*_*ST*_ distribution. The left and right dashed lines represent 20% left and right tails of the θ_π_ ratio distribution. The red points were identified as selected regions for high-altitude genotypes.

Genome regions with low nucleotide diversity were considered to be potential selection signatures from human domestication or different natural environment factors. A survey of meteorological information among these studied genotypes within a span of 32 years (from 1979 to 2010) showed significant correlations among UV-B, surface pressure, and altitude (Figure 2A). Sixty-six wild genotypes originated from two extreme habitats were used for a selective sweep analysis, of which 38 genotypes originated from high-altitude regions (>3,000 m) and 28 genotypes originated from low-altitude regions (< 1,000 m; Figure 2B). Two climate factors were significantly different between the two groups (*p* < 0.001, Welch’s test). The mean monthly cumulative UV-B value was 1535.9 and 2036.0 kJ⋅m^–2^ in low and high altitudes, respectively. Correspondingly, the mean surface pressure was 92.9 and 67.0 kPa, respectively. For the selection signals, a total of 965 SNPs were identified from the top 20% θ_π_ ratio (θ_π_, low altitude/θ_π_, high altitude) and the top 5% *F*_ST_ value for high-altitude samples (Figure 2C and Supplementary Table 3). Considering the characteristics of SLAF-based molecular markers in this study, the range where the distance between two adjacent markers is less than 100 kb and the distance span is greater than 100 bp was deemed as the selected area. We found 54 selected regions, of which the largest one was on chromosome 2A with eight SNPs and 255.7 kb of physical distance from 459,245,966 to 459,501,653 bp. The mean length was 16.1 kb (Supplementary Table 3). Seventy-three genes in response to harsh climate (such as low oxygen and strong ultraviolet radiation) in high-altitude areas were identified, including UV-B receptor UVR8 (3 genes), response to UV-B (5 genes), regulation of photosynthesis (9 genes), photosystem II (PSII) assembly (9 genes), chlorophyll (9 genes), carotenoid (2 genes), response to superoxide (1 gene), reactive oxygen metabolic process (1 gene), hydrogen peroxide (12 genes), peroxidase (15 genes), proline (7 genes), flavonoid biosynthetic process (7 genes), anthocyanin biosynthetic process (9 genes), L-ascorbic acid biosynthetic process (3 genes), and so on (Supplementary Table 4). Three haplotype regions with 17 (chr7B, from 267,243,758 to 267,243,793 bp), 8 (chr6A, from 609,091,993 to 609,092,385 bp), and 8 (chr1D, from 81,295,211 to 81,295,288 bp) close SNPs were annotated to gene functions of PSII assembly, anthocyanidin and chlorophyll biosynthetic process, and peroxidase activity, respectively. Five peroxidase activity-related genes were detected within 100-kb flanking regions on a chromosome 2B SNP (793,439,312 bp). Similar genes were found on chr2D (643,719,897 bp).

**FIGURE 3:**
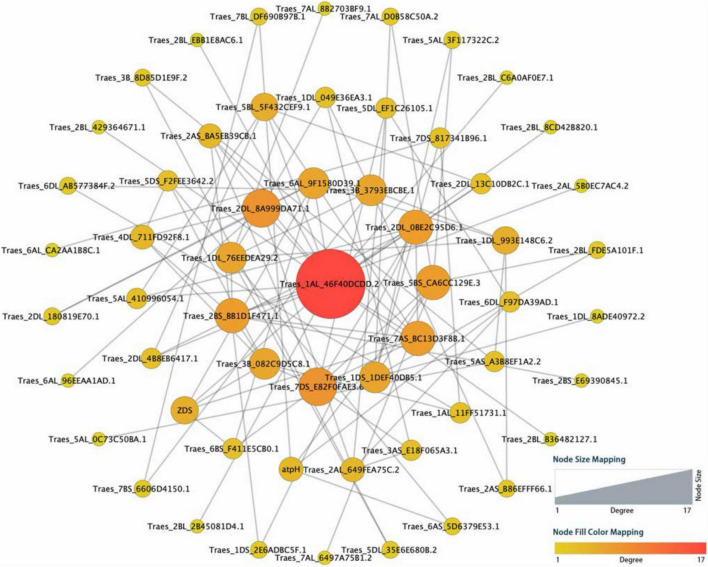
Protein–protein interaction (PPI) regulatory network for the selected candidate genes among high-altitude genotypes. The circles represented queried proteins, and the lines represented protein–protein associations.

To find out potential hub candidate genes responding to the harsh climate, a PPI network consisting of 57 nodes and 196 lines was obtained by using the online STRING database (Figure 3 and Supplementary Table 4). The analysis showed a tight PPI network, suggesting that the queried proteins have strong relationships and interactions. There were seven hub genes with the node degree greater than seven, including one with 17 degrees (*TraesCS1A01G442300*), two with 8 degrees (*TraesCS2D01G379400* and *TraesCSU01G146500*), and four with 7 degrees (*TraesCS2D01G462700*, *TraesCS5A01G554500*, *TraesCS5D01G065700*, and *TraesCS7B01G136000*). These genes are mainly involved in the response to UV light, flavonoid biosynthetic process, anthocyanin accumulation, reactive oxygen species (ROS) metabolic process, and cytochrome P450. Furthermore, the potential hub genes were important homologous genes (such as *CYP75B3*, *FabZ*, and *GPA1*) in Poaceae plants, including *Aegilops tauschii*, *Hordeum vulgare*, *Lolium rigidum*, and *Oryza sativa*, and may be important components in the genetic basis of the adaptation to high altitudes for *E. sibiricus*.

### Phenotypic Variation and Correlation Analysis

Four leaf-related traits: FLL, FLW, LL, and LW, four culm-related traits: PH, CD, TN, and CN, and four seed-related traits: SL, LS, WS, and SW1 were investigated within 2 years. A wide range of phenotypic variations were found among 210 *E. sibiricus* genotypes. The mean coefficient of variation (CV) between 12 phenotypic traits was 28.2%, varying from 6.8% (LS in 2018) to 84.1% (TN in 2018), and the average Shannon’s diversity index (H’) was 2.008 varying from 1.798 (TN in 2018) to 2.095 (WS in 2018; Table 2).

**TABLE 2:** Phenotypic variation statistics of genome-wide association study (GWAS) population.

Traits		Year	Min	Max	Mean	SD	CV (%)	H’^a^
Leaf-related	FLL(cm)	2017	5.5	27.3	13.9	4.6	32.9	1.921
		2018	5.0	19.6	10.5	2.6	25.3	1.943
	FLW(mm)	2017	3.05	16.70	6.18	2.0	32.5	1.870
		2018	3.42	11.94	7.25	1.7	23.2	2.055
	LL(cm)	2017	5.5	27.3	15.2	4.2	28.0	2.030
		2018	7.8	23.6	15.3	2.9	18.9	2.059
	LW(mm)	2017	2.77	11.99	6.17	1.8	29.2	1.971
		2018	3.48	9.32	6.07	1.4	22.9	2.073
Culm-related	PH(cm)	2017	12.2	73.2	31.4	12.3	39.1	1.917
		2018	17.3	101.5	55.0	19.5	35.5	2.035
	CD(mm)	2017	0.74	2.58	1.48	0.3	21.5	2.065
		2018	1.10	3.33	2.08	0.4	18.5	2.053
	TN(No.)	2017	3	152	42	26.4	62.2	1.936
		2018	2	310	66	55.6	84.1	1.798
	CN(No.)	2017	1	6	2	0.7	28.7	2.032
		2018	1	6	3	0.9	27.4	2.029
Seed-related	SL(cm)	2017	5.4	23.7	13.4	4.1	31.0	2.026
		2018	6.4	29.1	15.9	3.5	22.2	2.041
	LS(mm)	2017	4.45	11.75	8.87	1.0	11.7	2.006
		2018	7.75	11.02	9.51	0.6	6.8	2.030
	WS(mm)	2017	1.13	1.69	1.42	0.1	8.2	2.074
		2018	1.25	1.81	1.49	0.1	7.3	2.095
	SW1(g)	2017	0.713	5.360	3.208	0.9	28.1	2.047
		2018	0.653	3.986	2.222	0.7	31.9	2.090
Mean							28.2	2.008

*^a^H’, Shannon’s diversity index.*

**FIGURE 4:**
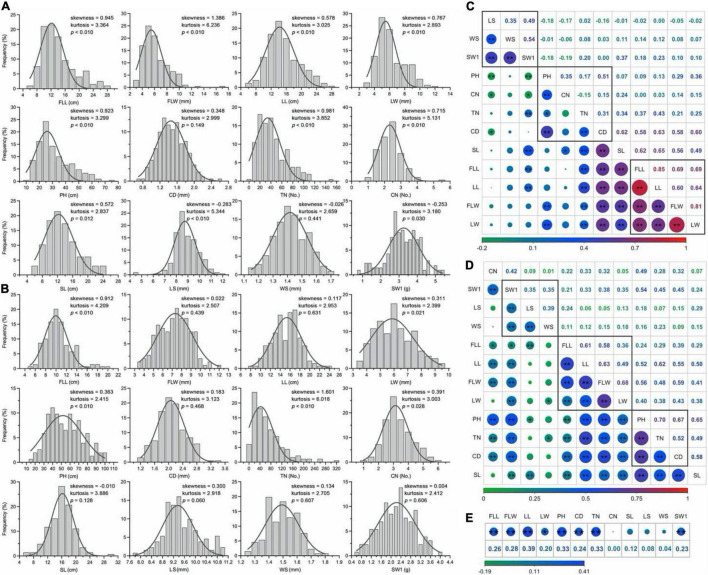
Distribution of phenotypic traits and correlation analysis in *E. sibiricus*. **(A,B)** Frequency distribution of the mean values of twelve phenotypic traits in 2 years. **(C,D)** Pair-wise Person correlations among different traits. The red to green color represents highly positive to highly negative correlations, and the number represents (upper diagonal) the correlation values. The lines in the square indicate the leaf-, culm-, and seed-related traits. Two (**) and single stars (*) indicate significance at 0.01 and 0.05, respectively. **(E)** Correlation analysis of twelve phenotypic traits between 2 years.

The phenotypic characters followed a normal or approximate normal distribution without any significant skewness and could be used for the subsequent analysis (Figures 4A,B). The Pearson’s correlation coefficient analysis showed significantly positive pairwise correlations between four leaf-related traits (FLL, FLW, LL, and LW; 0.60 to.85 in 2017 and.36 to.68 in 2018), as well as three seed-related traits (LS, WS, and SW1; 0.35 to.54 in 2017 and.35 to.39 in 2018; Figures 4C,D). These traits may be controlled by similar genetic factors. Two culm-related traits (TN and CD) had a highly significant positive correlation with leaf-related traits, of which the mean phenotypic correlation coefficients of.32,0.60 in 2017 and.44,0.49 in 2018, respectively. Generally, there were weakly positive or negative relations between two seed traits (LS and WS) and two grass yield-related traits (leaf and culm). Eight traits (FLL, FLW, LL, LW, PH, CD, TN, and SW1) had a higher correlation between two consecutive years (Figure 4E).

### Genome-Wide Association Analysis Reveals Loci Related to Agronomic Traits

**FIGURE 5:**
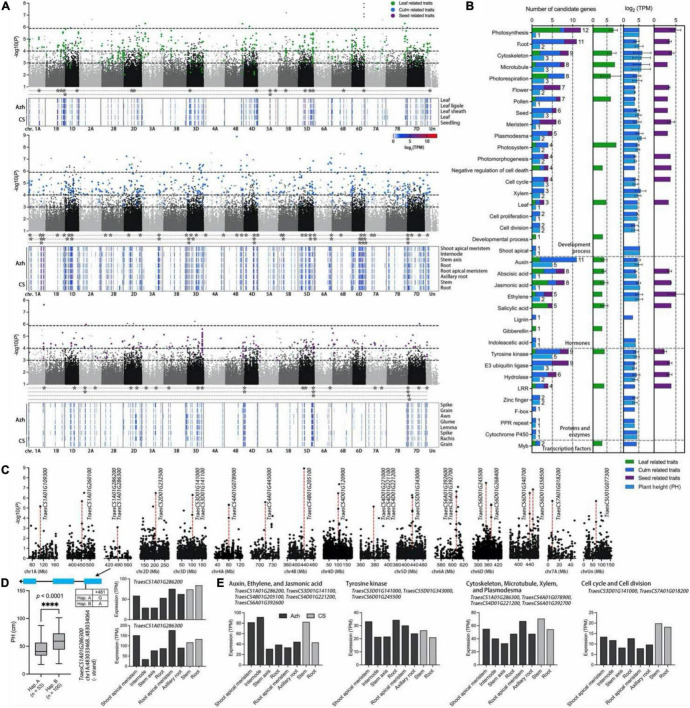
Genome-wide association study for phenotypic traits and identification of the candidate genes for the significant SNPs. **(A)** Manhattan plot for leaf (green points)-, culm (blue points)-, and seed (purple points)-related traits in *E. sibiricus*, respectively. Only SNPs with *P* values smaller than.1 are plotted. Three horizontal dashed lines represent *P* values at of 1.13e-6, 1e-4, and 1e-3, respectively. The gene expression profiles of Azhurnaya and Chinese Spring in leaf-, culm-, and seed-related traits were shown on the heatmap. The stars (*) represent the candidate genes with an expression (TPM) value of more than 10. **(B)** Frequency of category annotations and average TPM value for candidate genes. The green, blue, purple, and wathet blue bars represent leaf, culm, seed, and plant height (PH) traits, respectively. **(C)** Local Manhattan plot for PH. The red dashed lines indicate the significant loci with the highest –lg(P) value. **(D)** Exon–intron structure and DNA polymorphism of *TraesCS1A01G286300*. The difference between the two haplotypes was shown on the boxplots and statistically analyzed based on Welch’s test for plant height (PH, 2018). The expression profiles of two candidate genes were shown on the bar plots. **(E)** The expression profiles of twelve candidate genes were identified from PH trait.

Using a MLM with correction of genetic structure (Q) and kinship (K) bias, the multiple GWAS tests (GEMMA, EMMAX, and TASSEL) were performed to confirm significant SNPs associated with twelve grass yield- and seed-related traits in *E. sibiricus* (Supplementary Figures 3–5). The quantile-quantile (QQ) plots indicated well-controlled population stratification by using MLM and provided reliability for association analysis. In this study, 1,825 SNP markers were detected above the critical threshold of –lg*P* ≥ 4 (EMMAX and TASSEL) and 3 (GEMMA), respectively (Table 3, Supplementary Table 5, and Figure 5A). More than half of the associated loci (917) were distributed on D subgenomes, while 477, 398, and 33 SNPs were on B, A, and Un, respectively. Three softwares identified a total of 848 SNPs for culm-related traits, followed by leaf-related traits (604 SNPs) and seed-related traits (373 SNPs). Especially, 358, 247, and 235 SNPs were related to PH, FLW, and TN, respectively. The TASSEL test showed that the percentage of phenotypic variance explained (*R*^2^) ranged from 7.62 to 31.85%, with an average of 15.57%.

**TABLE 3:** Genome-wide association study analysis for twelve phenotypic traits using a mixed linear model (MLM) based on three algorithms.

Traits	Year	GEMMA					EMMAX					TASSEL						NJ^g^
						
		NS^a^	MP^b^	LP^c^	NAS^d^	CG^e^	NS	MP	LP	NAS	CG	NS	MP	LP	R^2f^	NAS	CG	
FLL(cm)	2017	18	9.35E–06	5.03	6	0	28	8.19E–07	6.09	12	1	38	6.29E-08	7.20	8.21–26.37%	16	1	3
	2018	30	6.05E–05	4.22	12	1	34	3.02E-06	5.52	12	0	13	1.10E-05	4.96	12.87–30.60%	7	1	5
FLW(mm)	2017	17	5.00E-07	6.30	8	0	92	7.05E–08	7.15	50	7	96	9.72E-09	8.01	8.58–30.94%	56	7	22
	2018	16	2.17E–05	4.66	9	0	21	1.51E–06	5.82	13	0	5	1.30E–05	4.89	13.63–26.13%	4	0	6
LL(cm)	2017	20	2.78E–06	5.56	9	1	22	2.79E–06	5.55	7	0	26	1.91E–06	5.72	9.44–30.21%	6	2	1
	2018	8	1.52E–04	3.82	4	0	12	4.95E–07	6.31	6	0	0	–	–	–	0	0	0
LW(mm)	2017	15	5.06E–06	5.30	5	0	25	1.21E–06	5.92	10	1	47	2.45E–07	6.61	9.22–23.85%	25	2	3
	2018	11	9.26E–05	4.03	3	0	10	3.57E–06	5.45	1	0	0	–	–	–	0	0	0
PH(cm)	2017	25	1.61E–05	4.79	12	1	33	1.80E–07	6.74	14	3	246	1.18E–09	8.93	7.62–30.9%	132	26	23
	2018	24	2.48E–06	5.60	13	5	18	3.15E–08	7.50	14	5	12	1.55E–05	4.81	10.74–27.32%	10	2	6
CD(mm)	2017	16	3.08E–05	4.51	5	3	13	1.84E–05	4.73	5	1	11	1.01E–05	5.00	7.82–14.90%	6	4	2
	2018	12	3.49E–07	6.46	2	1	20	4.69E–07	6.33	7	2	10	3.04E–06	5.52	11.13–23.49%	8	2	4
TN(No.)	2017	17	2.17E–06	5.66	3	3	33	8.78E–08	7.06	15	3	6	1.40E–06	5.86	13.33–22.86%	3	1	2
	2018	17	2.24E–06	5.65	7	1	61	3.68E–07	6.43	25	0	101	5.22E–07	6.28	9.33–29.14%	45	5	12
CN(No.)	2017	14	6.36E–06	5.20	10	1	38	1.74E–07	6.76	21	3	13	3.99E–06	5.40	15.30–27.92%	7	0	4
	2018	22	4.92E–07	6.31	11	1	53	1.21E–07	6.92	27	4	33	7.55E–07	6.12	12.02–31.85%	19	0	2
SL(cm)	2017	12	3.99E–05	4.40	7	1	19	8.81E–07	6.05	16	2	0	–	–	–	0	0	3
	2018	23	2.36E–05	4.63	12	0	21	8.72E–07	6.06	11	2	4	2.11E–05	4.68	12.11–27.29%	2	0	1
LS(mm)	2017	23	3.09E–05	4.51	17	1	45	3.00E–06	5.52	27	2	45	8.41E-07	6.08	9.94–24.16%	29	9	12
	2018	50	2.54E–05	4.59	25	5	11	1.80E–05	4.74	9	0	0	–	–	–	0	0	1
WS(mm)	2017	24	3.97E–05	4.40	9	2	8	4.46E–06	5.35	6	1	7	2.88E-05	4.54	10.26–17.30%	4	0	3
	2018	13	3.47E–05	4.46	8	1	5	4.06E–06	5.39	1	0	2	1.28E–05	4.89	22.10–30.58%	1	2	0
SW1(g)	2017	21	5.49E–07	6.26	10	0	24	2.38E–08	7.62	5	1	1	2.18E–05	4.66	12.99–12.99%	1	0	1
	2018	8	8.54E–05	4.07	4	0	7	1.88E–05	4.73	2	3	0	–	–	–	0	0	0
Sum		456			211	28	653			316	41	716				381	64	116

*^a^Number of associated SNPs.*

*^b^The min value of P.*

*^c^The peak value of -lgP.*

*^d^Number of annotated SNPs.*

*^e^Number of candidate genes.*

*^f^Percentage of variance explained.*

*^g^Number of SNPs jointly annotated by more than two software.*

A gene function annotation analysis was performed to rapidly identify the candidate genes associated with yield-related traits in *E. sibiricus*. There were 1,064 genes identified from 908 significant SNPs within 100-kb flanking regions, of which 344 loci with multigenic effects (Supplementary Table 6). In the TN traits, a SNP (6,982,833) on chromosome 1D associated with 8 genes related to the process of recognizing pollen, response to growth hormone, tyrosine kinase, ankyrin repeats, and leucine-rich repeat (LRR). Eight closely linked genes identified from a SNP marker on chr1D (37,459,155), annotated with plasmodesma, cytoskeleton, F-box protein, PPR repeat family, and hydrolases family, may result in multigenic effects for plant height traits. A total of 116 highly consistent SNPs that overlapped from more than two softwares were deemed to be highly reliable SNPs. After a comparison of associated genes with gene expression data of two wheat cultivars (Azhurnaya and Chinese Spring), we finally identified 106 candidate genes (Figure 5A). Sixty-two genes were related to PH, CD, TN, and CN traits. Additionally, 20 and 28 genes were related to leaf- and seed-related traits, respectively. Furthermore, functional classifications of these genes were mainly involved in four enriched biological processes: plant development process (70 genes), protein and enzymes (30 genes), hormones (26 genes), and transcription factors (2 genes; Figure 5B). The functional annotation showed that the biological process of photosynthesis, root, cytoskeleton, microtubule, photorespiration, flower, pollen, and seed were the most highly represented categories in the plant development process. For the biological regulation of hormone signals, many terms were enriched with auxins, abscisic acid (ABA), jasmonic acid, ethylene, and salicylic acid. Tyrosine kinase, E3 ubiquitin ligase, hydrolase, and LRRs were the major proteins and enzymes. For leaf-related traits, the top four gene functions were photosynthesis, photorespiration, jasmonic acid, and salicylic acid, whereas E3 ubiquitin ligase, meristem, flower, and photosynthesis were enriched for seed-related traits. The candidate genes for culm-related traits covered almost all molecular functional categories, and microtubule, auxins, tyrosine kinase, and E3 ubiquitin ligase were major enrichment terms.

For the PH traits, 40 SNPs with 38 candidate genes were identified (Figure 5C and Supplementary Table 6). The GEMMA test found a SNP marker (chromosome 1A, 483,033,948) on exon region of *TraesCS1A01G286300*, a plasmodesma-related gene, and the non-synonymous SNP diversity caused amino acid differences between Methionine (Met) and Isoleucine (Ile; Figure 5D). The haplotype-based analysis showed that the Hap. B (SNPs of A) with 100 genotypes was significantly positively correlated with plant height (PH; *p* < 0.0001, Welch’s test) which increased by 32.7% when compared with 53 genotypes with Hap. A (SNPs of G; Figure 5D). In addition, the gene was highly expressed in the internode, stem axis, and stem of two wheat cultivars, respectively. Furthermore, these genes showed four main functional annotations: (1) ethylene, auxins, and jasmonic acid signaling pathway; (2) tyrosine kinase activity; (3) cytoskeleton organization, microtubule, xylem formation, and plasmodesma; (4) cell cycle and cell division (Figure 5E). A total of 12 genes were highly overexpressed across all culm-related traits. Particularly, two SNPs on chromosome 4A (80,134,459) and 6D (378,425,315) were jointly annotated by GEMMA, EMMAX, and TASSEL. A gene *TraesCS4B01G205100* had a higher expression level in internode and stem in two wheat cultivars, which was located within the 100-kb flanking regions of significant loci with the highest –lg(*P*) value (8.929) in the GWAS test.

**FIGURE 6:**
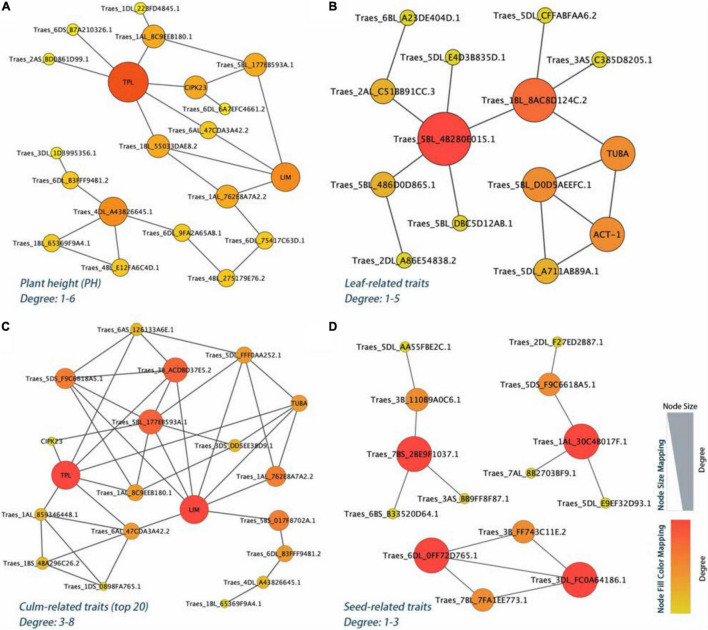
The PPI regulatory network for the yield-related candidate genes in *E. sibiricus*. **(A)** Plant height (PH) traits. **(B–D)** Leaf-, culm-, and seed-related traits, respectively.

The PPI network analysis further narrowed down the list of 106 identified yield-related candidate genes in *E. sibiricus*. Based on the degree algorithm, there were three hub genes, *TraesCS4D01G221200* (TPL), *TraesCS4D01G221100* (Traes_4DL_A43826645.1), and *TraesCS7D01G194800* (LIM), with the degree value more than 4 detected within PH traits (Figure 6A and Supplementary Table 7). The top gene, *TraesCS4D01G221200*, was found to be involved in the biological regulation process of a variety of plant hormones and morphological development, including auxins- and jasmonic acid-mediated signaling pathway; meristem and shoot apical meristem (SAM) specification; xylem and phloem pattern formation. Besides, two (*TraesCS5A01G457500* and *TraesCS1B01G254000*), six (*TraesCS4D01G221200*, *TraesCS7D01G194800*, *TraesCS3D01G190400*, *TraesCS5D01 G343000*, *TraesCS1A01G286200*, and *TraesCS5D01G118000*), and four (*TraesCS1A01G402400*, *TraesCS3D01G296900*, *TraesCS6A01G307700*, and *TraesCS7D01G056800*) hub genes were detected in leaf-, culm-, and seed-related traits, respectively (Figures 6B–D). These genes mainly played the biological function of photosynthesis (leaf-related traits); auxins, jasmonic acid, and ethylene-mediated signaling pathway, meristem, shoot, and xylem apical meristem specification, cell proliferation, tyrosine kinase and E3 ubiquitin-protein ligase activity (culm-related traits); and tyrosine kinase, flower development, photosynthesis, and cytoskeleton organization (seed-related traits).

## Discussion

### Selective Signature Detection in *Elymus sibiricus* at High Altitudes

In this study, we performed SLAF-seq of 210 *E. sibiricus* genotypes including six geographical populations from the main distribution areas of the species. A total of 88,506 high-consistent SNPs were picked up for genetic research. Phylogenetic relationship and population architecture analysis revealed that the Geo_1 group collected from Qinghai-Tibet Plateau (QTP) regions had different genetic backgrounds from the other groups (Figures 1A,B,D,E). A similar result was supported by some genetic diversity studies in *E. sibircus*, including chromosome karyotype analysis, gliadin, and SSR marker study (Ma et al., 2012b; De et al., 2014; Yan et al., 2017). It may indicate that the main climatic factors act as a selective pressure on different geographical populations. The QTP population experienced severe natural pressure to adapt to harsh environmental factors in high-altitude regions, such as low pressure, strong radiation, sharp temperature shifts, and overwintering challenges. To understand the genetic bases of adaptation to high plateaus from a genome perspective, two pools of *E. sibiricus* genotypes collected from high-altitude regions (>3,000 m) and low-altitude regions (<1,000 m) were used to detect the associated genes for altitude adaptation. More than 70 genes, mainly responding to UV-B and superoxide, UV-B receptor UVR8, regulating photosynthesis, PSII, flavonoid biosynthetic process, and peroxidase activity, were identified among 965 strong selected loci. Among them, seven genes were at the hub position in the PPI network analysis, which play pivotal roles in response to UV light, flavonoid biosynthetic process, anthocyanin accumulation, ROS metabolic process, and cytochrome P450.

The selective filter of sunlight in leaf epidermal tissues represented an acclimatizing mechanism by which plants are exposed to strong UV radiation conditions (including UV-B, 280–315 nm) for a long time (Barnes et al., 2016). In response to UV radiations, the common stress-related processes include antioxidant defense and accumulation of UV-absorbing compounds in the vacuoles and cell walls of leaf epidermal cells, such as flavonoids, anthocyanin, and other phenolics, is activated (Siipola et al., 2015; Wu Q. et al., 2016; Berland et al., 2019). Besides, the photomorphogenic responses, gene expression, and accumulation of metabolites were triggered by UV RESISTANCE LOCUS 8 (UVR8), a specific UV-B photoreceptor in plants (Rizzini et al., 2011). In *Arabidopsis*, transgenic plants with UVR8 showed elevated levels of anthocyanin and enhanced the acclimation-independent UV-B tolerance (Heijde et al., 2013; Podolec et al., 2021). UV-B induced damage to photosynthetic machinery and limit of PSII activity inhibits growth and biomass accumulation, especially in UV-B sensitive plants. It was reported that ambient UV radiations reduced leaf area by 23% in *Lactuca sativa* L. in mid-latitude locations (Wargent et al., 2011). *E. sibiricus* can thrive in high-alpine areas and shows good tolerance. However, the information related to the effects of ultraviolet radiations on *E. sibiricus* growth among different habitats is limited; thus, future exploration of UV light adaptation mechanisms may provide profound benefits to increase the forage grass production. Furthermore, exposure to UV-B among alpine plants can result in cross-protection against other stress factors at the same time, such as low temperature and hypoxic conditions on Qinghai-Tibet Plateau; thus, a combined stress environment should be considered (Wargent et al., 2011; Schulz et al., 2021). The comprehensive ecological environment on Qinghai-Tibet Plateau drove the adaptation strategies in *Leymus secalinus* and activated antioxidant defense system with multiple protective strategies, including the reduction of chlorophyll contents and increase in carotenoid content to protect plants against photodamage; the changes in antioxidant enzyme activities enhanced the ROS scavenging capacity; the increases in proline and soluble sugar contents adjust osmotic in plants of alpine regions (Cui et al., 2018). In this study, seven hub genes were all related to the antioxidant defense system and may play a key role in the adaptation strategies of *E. sibiricus*. As the significant ambient difference existed between the two groups, the genetic signatures and functional genes we identified may be preferentially amplified to respond to specific environment.

### Genome-Wide Association Study in *Elymus sibiricus*

Recently, advances in next-generation sequencing have rapidly increased the density of high-quality molecular markers in many non-model plants (Fang et al., 2017a; Pang et al., 2020; Shen et al., 2020; Wei et al., 2021). As the gigantic genome existed in *E. sibiricus*, with an estimate of 6.86 Gbp, we used a mature reduced whole-genome sequencing technology (SLAF-seq) to investigate the SNP information among 210 genotypes (Sun et al., 2013; Xiong et al., 2021). At present, there is no report on the assembled genome; thus, generated SNPs were mapped on the closest wheat reference genome (v1.0), with 79% of homogeneity and 84.67% of mapping rate of reads (Zhang et al., 2019a). Our study generated a total of 88,506 highly consistent SNPs, with an average sequencing depth of 18.01-fold and a marker density of 211.5 kb per SNP. Generally, the LD decay in cross-pollinated plants is much faster than in self-pollinated. A precise LD decay distance in *E. sibiricus* was unavailable in the study, but some self-pollinated species provided certain reference information. The physical distance of LD decay in rice, cottons, wheat, and soybean occurred at 445 kb (*r*^2^ = 0.2; Yano et al., 2016), 1,000 kb (*r*^2^ = 0.3; Fang et al., 2017b), 4.4 Mb (*r*^2^ = 0.5; Pang et al., 2020), and 223.2 kb (*r*^2^ = 0.4; Sui et al., 2020), respectively. Similarly, the mean distance between the markers was very less than the LD decay distances in our study, and SNP set provided sufficient resolution for capturing the genetic variation in general GWAS.

The identification of genes associated with important agronomic traits such as biomass and quality in forage grass is indispensable for breeding improvement (Yano et al., 2016). Previously, we identified 29 QTLs of seed-related traits among 14 linkage groups in *E. sibiricus* by using a biparental allelic separation population (F_2_; Zhang et al., 2019a). However, the restrictions with limited allelic segregation and recombination in linkage mapping, low genome coverage of molecular markers, low resolution of QTL regions, and small effects of polygenic interaction had hindered the effective identification of critical genes. The GWAS can detect the relationship between natural allelic variations and phenotypic variation and can provide an effective strategy for the rapid identification of key genes responsible for complex quantitative traits. The method overcomes the limitation of biparental populations and offers more variation sites from historical recombination events. The diversity panel of germplasm resources provides abundant variation sites for GWAS. In this study, the PCA and NJ-tree analyses showed a continuous distribution without a strong population structure, and the genotypes we selected were helpful to control false positives in GWAS. The MLM further controlled the population stratification and familial kinship of the accessions and is extensively used for crops and forage grass research as a popular method (Li H. et al., 2013; Valluru et al., 2017; Ramstein et al., 2018; Kang et al., 2019; Chen et al., 2021; Li et al., 2021). In addition, high-throughput sequencing generated more than tens of thousands of markers, and many loci have strongly LD against the estimation of false-positive rate by using independent tests among the actual number of markers in the Bonferroni assumption (Li et al., 2012; Li H. et al., 2013). A secondary identification of GWAS signals using an enlarged candidate region, considering the deviation of observed *P*-values from expected distribution in quantile-quantile (Q-Q) plots, increased some of the false-negative SNPs filtered by too strict and conservative Bonferroni correction (Valluru et al., 2017; Li et al., 2019; Mandozai et al., 2021).

### Candidate Genes for Yield-Related Traits

Based on the phenotypic observation and joint analysis with three programs, the GWAS efficiently detected a total of 1,825 significant loci associated with 12 grass yield- and seed-related traits in *E. sibiricus*. To evaluate the effects of identified genes, each of these was used to analyze the gene expression profiles, and we finally generated 106 candidate genes with higher expression levels (TPM values > 10) and indicated different functions (Figure 5). For example, 7 photosynthesis genes and 4 photorespiration genes were highly expressed in plant leaves. Four genes involved in plant hormone salicylic acid and 4 genes involved in jasmonic acid play an important role in plant growth and development, as well as plant photosystems in response to light stress (Nitschke et al., 2016; Chen et al., 2020). Furthermore, the PPI network analysis was employed to focus on the hub genes related to yield-related traits in *E. sibiricus* (Figure 6).

For PH traits, the top hub gene, *TraesCS4D01G221200*, is involved in a variety of biological regulation processes, such as auxins- and jasmonic acid-mediated signaling pathway, SAM, xylem, and phloem pattern formation. Meanwhile, the corresponding protein of this gene, TOPLESS (TPL), a conserved co-repressor, regulates a wide array of plant physiology process as central to multiple signaling pathways in higher plants, such as development, meristem maintenance, and hormone signaling (auxins, jasmonic acid, and ethylene; Causier et al., 2012; Wang et al., 2013). During post-embryonic development, the shoot and the root apical meristems are responsible for the formation of the main axis of plant growth. In *Arabidopsis* embryogenesis, the *topless-1* (*tpl-1*) mutation failed to form a SAM, for the absence of repression system (TPL), which acts to the expression of root-promoting genes in the top half of the embryo (Long et al., 2006). Cell-to-cell communication is a prerequisite for the process of growth and development for multicellular organisms. Plasmodesmata (PD) connects the adjacent individual cells to the entire organism, allowing transduction and exchange of diverse macromolecules, metabolites, proteins, nutrients, and phytohormones during plant growth and development (Vu et al., 2020). Mutants of multiple genes have been shown to be responsible for PD function. At the stage of embryogenesis in *Arabidopsis*, *ise1* and *ise2* mutants increased the PD-mediated transport and the PD permeability (Kobayashi et al., 2007; Stonebloom et al., 2009; Carlotto et al., 2016). Whereas, *dse1* mutant reduced the transport *via* PD and exhibited developmental defects at all development stages including smaller plants and delayed flower initiation than the wild type (Xu et al., 2012). Induction of a synthetic allele *icals3m* can effectively block the movement of proteins and RNAs *via* PD by increasing callose and decreasing the size of PD aperture. In root meristem of *Arabidopsis*, this interference in cell-to-cell communication significantly caused the loss of symplastic signaling of cells and tissues around and is critical for the regulation of cell divisions and cell expansion (Wu S. et al., 2016). In our study, we associated a non-synonymous SNP within the exon coding region of *TraesCS1A01G286300*, which was functionally related to plasmodesma. Our data showed a phenotype difference of 32.7% (PH) in the two haplotypes. The gene that was highly expressed in the internode, stem axis, and stem of two wheat cultivars may be involved in the development process in *E. sibiricus* and caused the trait differences.

In addition, the candidate genes which encode auxin signaling (9 genes), tyrosine kinase (6 genes), E3 ubiquitin ligase (5 genes), microtubule (6 genes), and cytoskeleton (5 genes) were largely involved in culm-related traits. Moreover, the genes related to the hormone signaling pathway, tyrosine kinase activity, cytoskeleton, and microtubule structure tend to be in the center position in the PPI analysis. As a multi-functional phytohormone, auxins modulate numerous plant processes including SAM development, vascular elongation, and axillary root formation (De Smet and Jürgens, 2007). The polar auxin transport was impaired in the maize mutant with qph1, a major plant height QTL, and decreased plant height by 20% (Xing et al., 2015). In addition, microtubule growth and cell cytoskeleton rearrangements have been shown to be accompanied by auxin regulation (Winnicki, 2020). In the meristematic cells of the root epidermis of *Arabidopsis thaliana*, the auxin signaling influences actin cytoskeleton, contributing to the vacuolar morphogenesis and leading to the regulation of cell size in plants (Scheuring et al., 2016). Furthermore, the plant receptors play a crucial regulatory role in many cell signaling pathways leading to cell growth, development, and disease resistance. Eukaryotic protein kinases (ePKs) facilitate the signal transduction and activation (or inactivation) of downstream reaction by phosphorylation of substrate protein of serine (Ser)/threonine (Thr)/tyrosine (Tyr) residues (Schwessinger et al., 2011; Jose et al., 2020). In model plants, rice and *Arabidopsis*, the proportions of conserved phosphorylation sites of serine and threonine were about 80–85% and 10–15%, respectively, while tyrosine was less than 5% (Nakagami et al., 2010). Despite lower abundance, Tyr phosphorylation has been demonstrated to be involved in different stages and processes of plant development, including the ABA pathway in seeds (Ghelis et al., 2008), leaf size, flowering time (Oh et al., 2009), plant growth (Jaillais et al., 2011; Nemoto et al., 2017), and root hair growth and development (Yemets et al., 2008).

## Data Availability Statement

The datasets presented in this study can be found in online repositories. The names of the repository/repositories and accession number(s) can be found in the article/Supplementary Material.

## Author Contributions

YW, WL, SB, and WX conceived the research. WL and SB provided the germplasm resources and reviewed the manuscript. ZZ, YZ, and NW implemented the experiment. ZZ, YZ, and JZ performed the data curation and analysis. ZZ and WX wrote the manuscript. All authors approved the final manuscript.

## Conflict of Interest

The authors declare that the research was conducted in the absence of any commercial or financial relationships that could be construed as a potential conflict of interest.

## Publisher’s Note

All claims expressed in this article are solely those of the authors and do not necessarily represent those of their affiliated organizations, or those of the publisher, the editors and the reviewers. Any product that may be evaluated in this article, or claim that may be made by its manufacturer, is not guaranteed or endorsed by the publisher.

## Footnotes

1
https://wheat-urgi.versailles.inra.fr/Seq-Repository/Publications-IWGSC

2
https://evolution.genetics.washington.edu/phylip.html

3
https://itol.embl.de/

4
http://www.cog-genomics.org/plink/1.9/

5
https://www.ecmwf.int/en/forecasts/datasets/browse-reanalysis-datasets

6
https://ftp.ncbi.nlm.nih.gov/blast/db

7
http://geneontology.org

8
http://pfam.xfam.org

9
https://ftp.ncbi.nih.gov/pub/COG/COG2020/data/

10
https://ftp.ncbi.nih.gov/pub/COG/KOG/

11
http://ftp.ebi.ac.uk/pub/databases/swissprot

12
https://www.kegg.jp/kegg

13
https://cn.string-db.org/

14
http://www.wheat-expression.com/

## Supplementary Material

Supplementary Figure 1 Distribution of single nucleotide polymorphisms (SNPs) in a window size of 5 Mb (**A**, with all of 13,643,515 SNPs) and 20 Mb (**B**, with 88,506 filtered SNPs) on 22 chromosomes of wheat. Each color represents different densities.

Supplementary Figure 2 The heatmap of kinship matrix of the 210 *Elymus sibiricus* genotypes.

Supplementary Figure 3 Genome-wide association study (GWAS) results of 12 phenotypic traits in *E. sibiricus* by GEMMA test. **(A)** Manhattan plots. The threshold was set as *P* < 9.36e-6 and 1e-3, respectively. **(B)** The Q-Q plots.

Supplementary Figure 4 GWAS results of 12 phenotypic traits in *E. sibiricus* by EMMAX test. **(A)** Manhattan plots. The threshold was set as *P* < 1.13e-6 and 1e-4, respectively. **(B)** The quantile-quantile (Q-Q) plots.

Supplementary Figure 5 Genome-wide association study results of 12 phenotypic traits in *E. sibiricus* by TASSEL test. **(A)** Manhattan plots. The threshold was set as *P* < 1.13e-6 and 1e-4, respectively. **(B)** The Q-Q plots.
