# Supplementary figures and images for: High-Altitude Genetic Selection and Genome-Wide Association Analysis of Yield-Related Traits in Elymus sibiricus L. Using SLAF Sequencing
Source: Front Plant Sci. 2022 Jun 21;13:874409. doi: 10.3389/fpls.2022.874409 (PMC9253694; doi:10.3389/fpls.2022.874409)

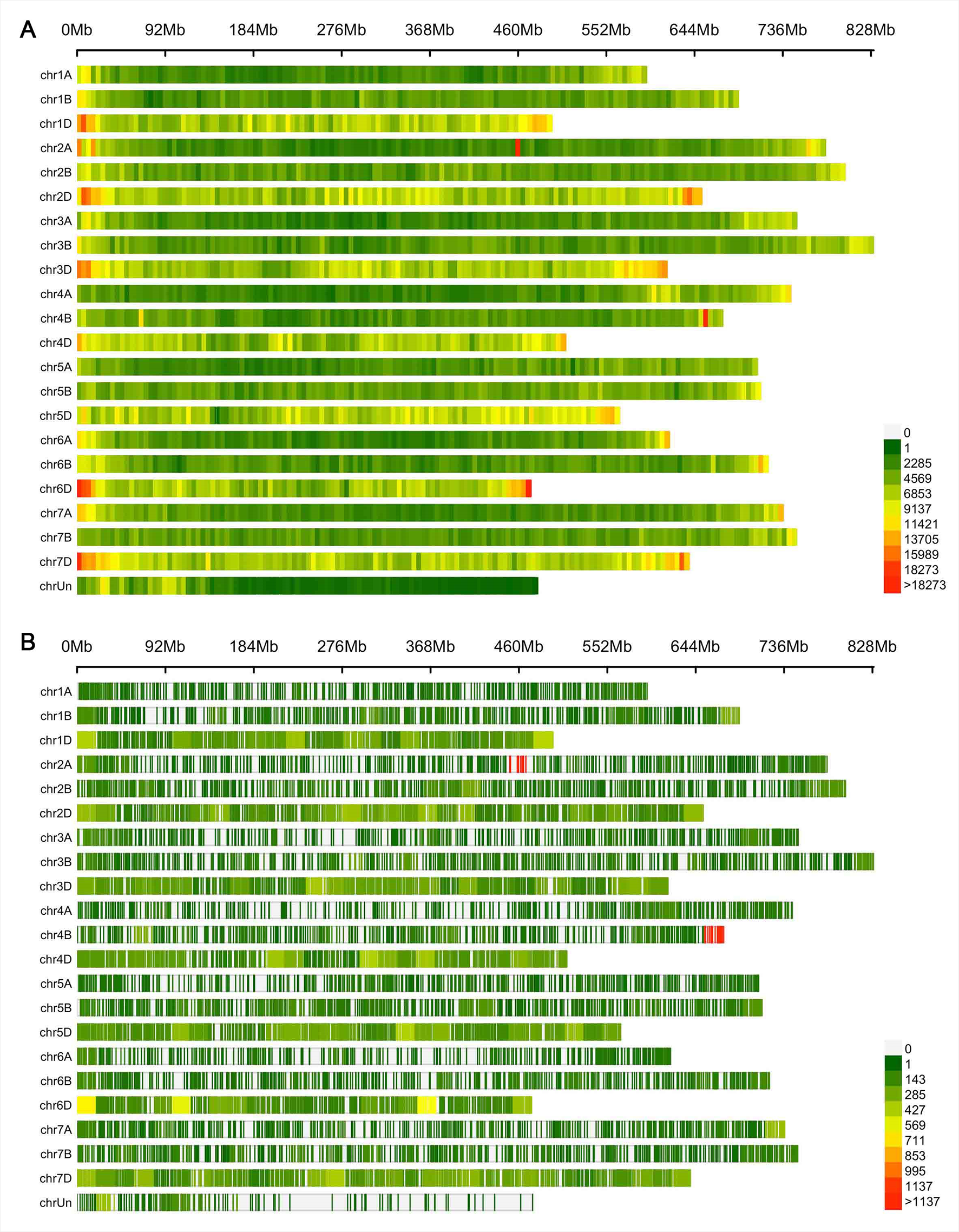

Supplement: Supplementary Figure 1 — Distribution of single nucleotide polymorphisms (SNPs) in a window size of 5 Mb (A, with all of 13,643,515 SNPs) and 20 Mb (B, with 88,506 filtered SNPs) on 22 chromosomes of wheat. Each color represents different densities. [file Image_1.TIFF]

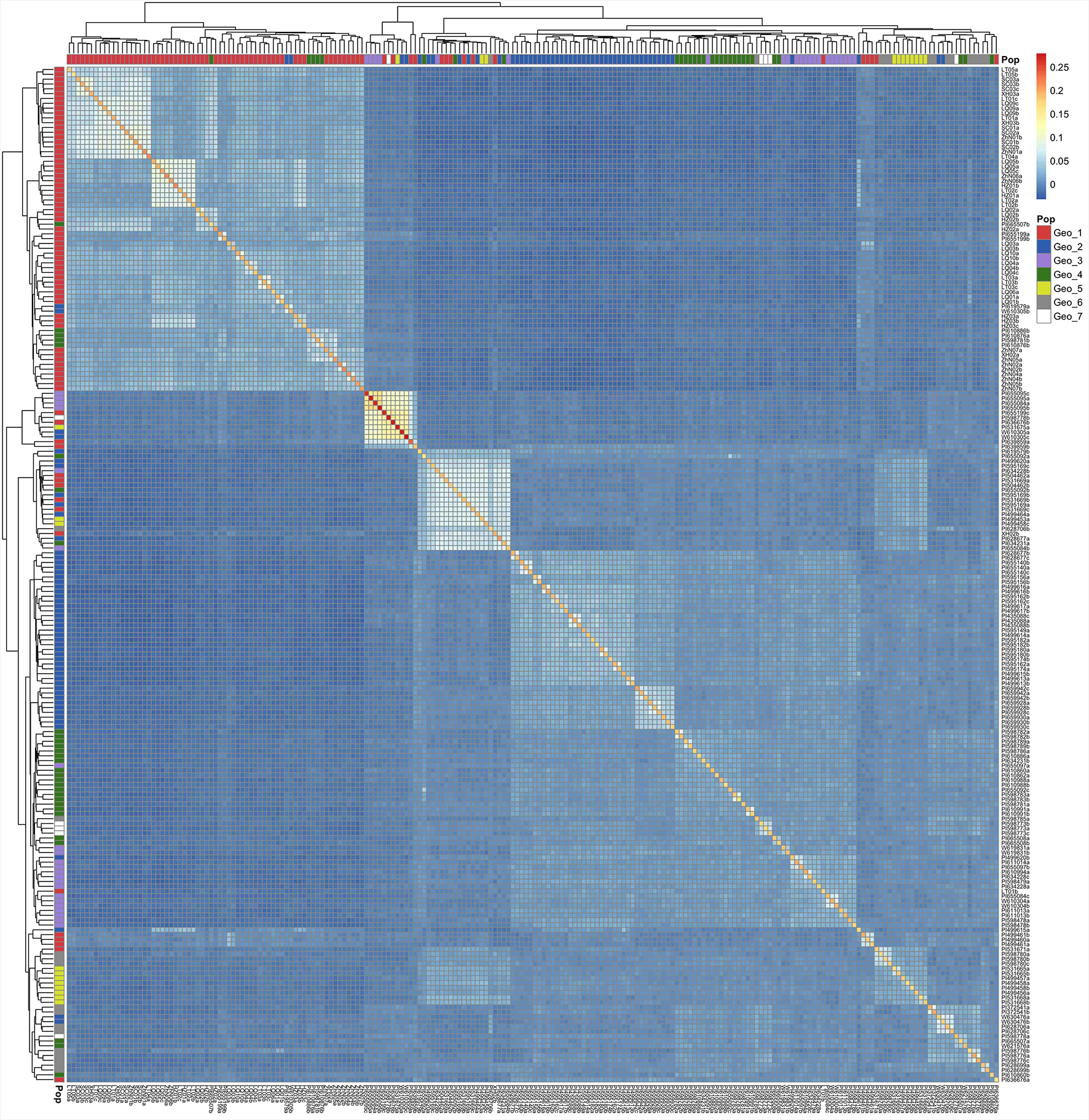

Supplement: Supplementary Figure 2 — The heatmap of kinship matrix of the 210 Elymus sibiricus genotypes. [file Image_2.TIFF]

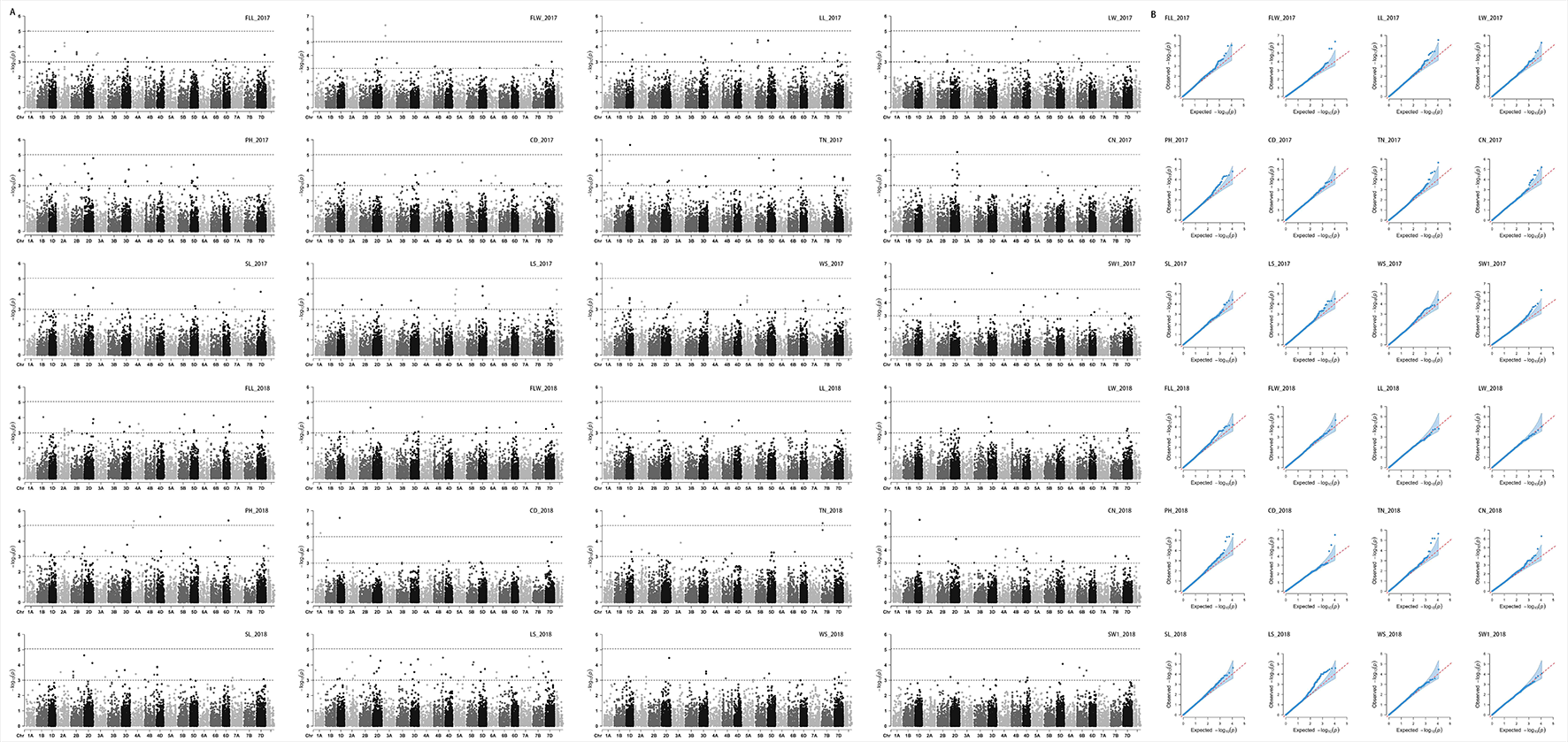

Supplement: Supplementary Figure 3 — Genome-wide association study (GWAS) results of 12 phenotypic traits in E. sibiricus by GEMMA test. (A) Manhattan plots. The threshold was set as P < 9.36e-6 and 1e-3, respectively. (B) The Q-Q plots. [file Image_3.TIFF]

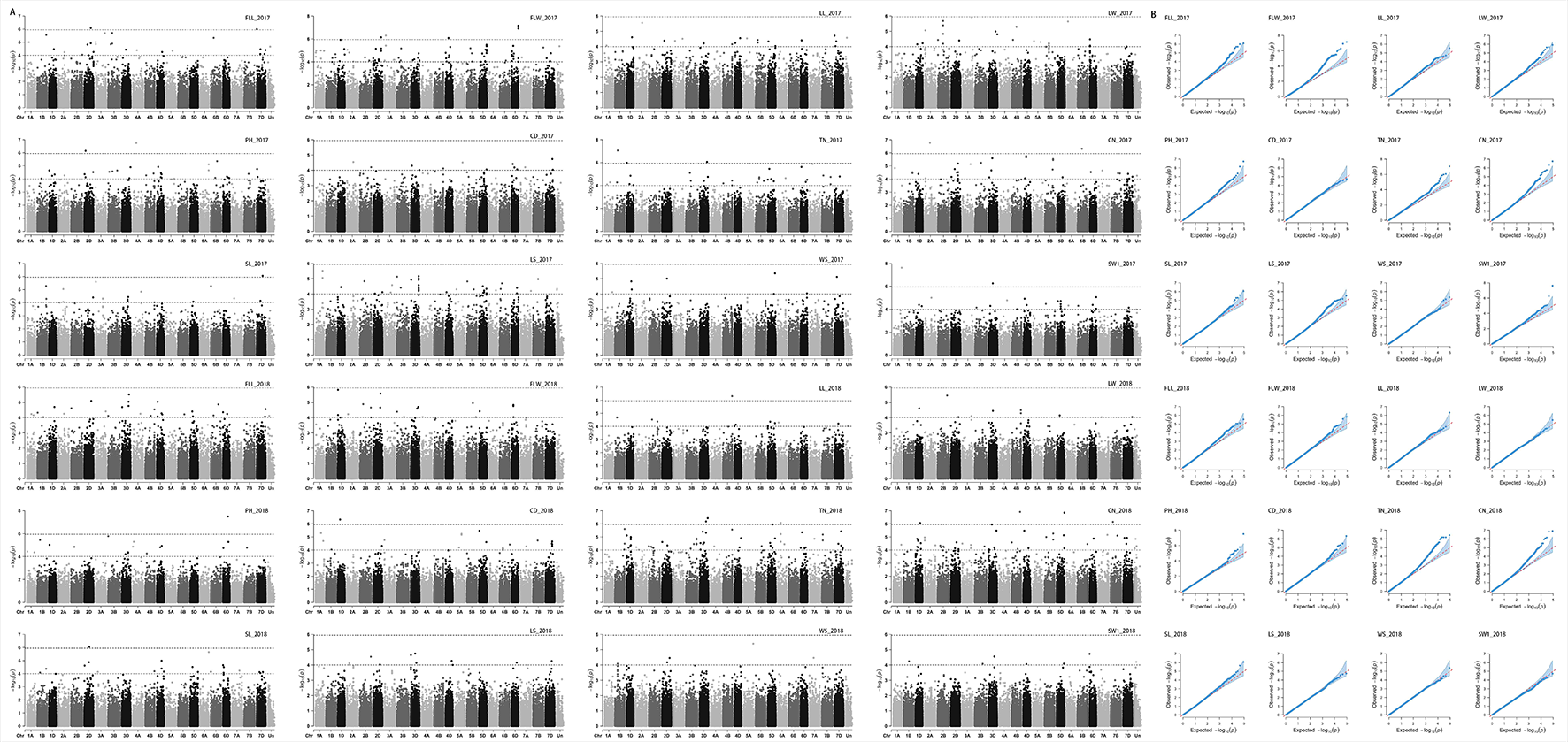

Supplement: Supplementary Figure 4 — GWAS results of 12 phenotypic traits in E. sibiricus by EMMAX test. (A) Manhattan plots. The threshold was set as P < 1.13e-6 and 1e-4, respectively. (B) The quantile-quantile (Q-Q) plots. [file Image_4.TIFF]

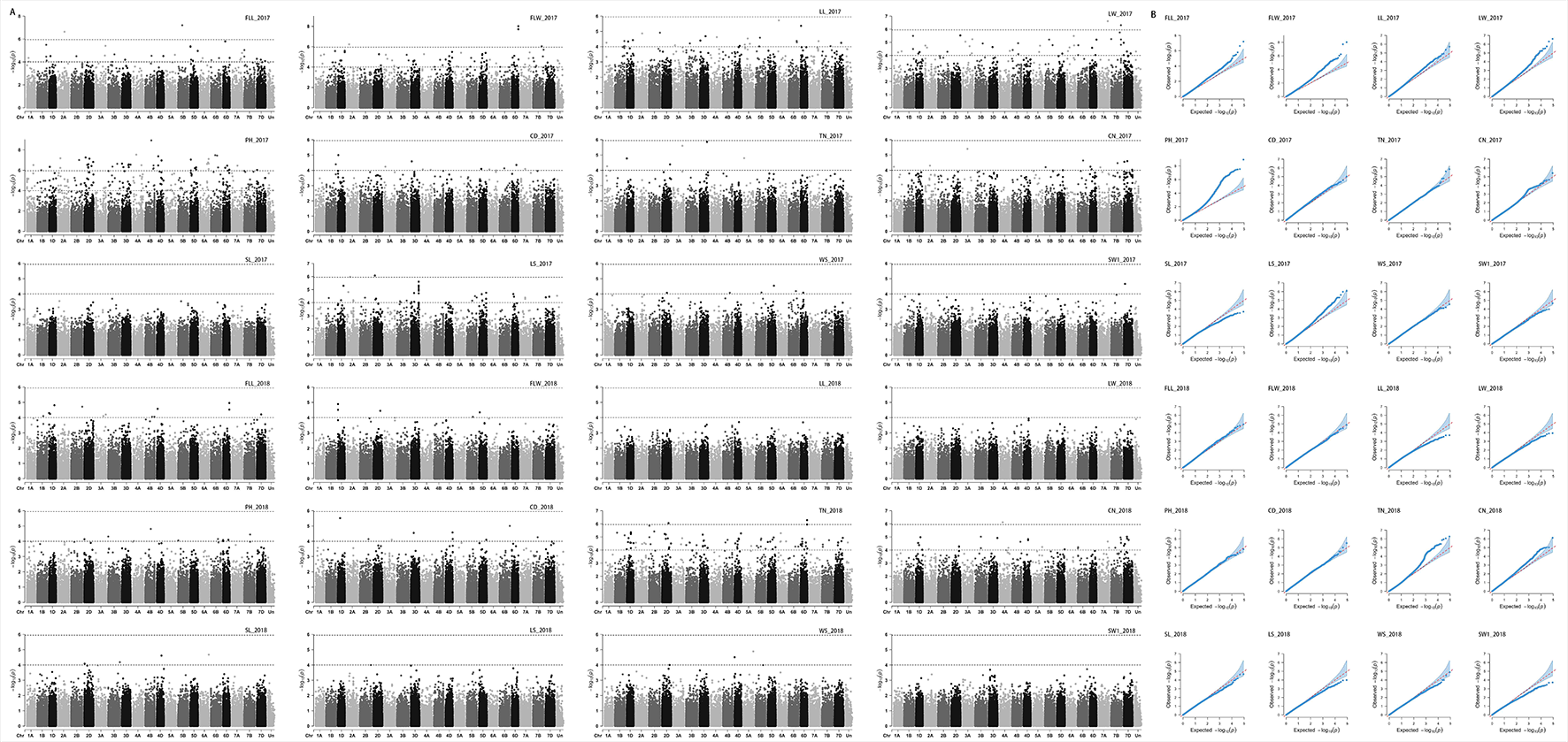

Supplement: Supplementary Figure 5 — Genome-wide association study results of 12 phenotypic traits in E. sibiricus by TASSEL test. (A) Manhattan plots. The threshold was set as P < 1.13e-6 and 1e-4, respectively. (B) The Q-Q plots. [file Image_5.TIFF]
